# Reprogramming of IL-12 secretion in the PDCD1 locus improves the anti-tumor activity of NY-ESO-1 TCR-T cells

**DOI:** 10.3389/fimmu.2023.1062365

**Published:** 2023-01-30

**Authors:** Segi Kim, Cho I Park, Sunhwa Lee, Hyeong Ryeol Choi, Chan Hyuk Kim

**Affiliations:** Department of Biological Sciences, Korea Advanced Institute of Science and Technology, Daejeon, Republic of Korea

**Keywords:** immunotherapy, TCR-T, interleukin-12, CRISPR/Cas9, NY-ESO-1, PD-1

## Abstract

**Introduction:**

Although the engineering of T cells to co-express immunostimulatory cytokines has been shown to enhance the therapeutic efficacy of adoptive T cell therapy, the uncontrolled systemic release of potent cytokines can lead to severe adverse effects. To address this, we site-specifically inserted the *interleukin-12* (IL-12) gene into the PDCD1 locus in T cells using clustered regularly interspaced short palindromic repeats (CRISPR)/CRISPR-associated protein 9 (Cas9)-based genome editing to achieve T-cell activation-dependent expression of IL-12 while ablating the expression of inhibitory PD-1.

**Methods:**

New York esophageal squamous cell carcinoma 1(NY-ESO-1)-specific TCR-T cells was investigated as a model system. We generated ΔPD-1-IL-12 -edited NY-ESO-1 TCR-T cells by sequential lentiviral transduction and CRISPR knock-in into activated human primary T cells.

**Results:**

We showed that the endogenous *PDCD1* regulatory elements can tightly control the secretion of recombinant IL-12 in a target cell-dependent manner, at an expression level that is more moderate than that obtained using a synthetic NFAT-responsive promoter. The inducible expression of IL-12 from the *PDCD1* locus was sufficient to enhance the effector function of NY-ESO-1 TCR-T cells, as determined by upregulation of effector molecules, increased cytotoxic activity, and enhanced expansion upon repeated antigen stimulation in vitro. Mouse xenograft studies also revealed that PD-1-edited IL-12-secreting NY-ESO-1 TCR-T cells could eliminate established tumors and showed significantly greater in vivo expansion capacity than control TCR-T cells.

**Discussion:**

Our approach may provide a way to safely harness the therapeutic potential of potent immunostimulatory cytokines for the development of effective adoptive T cell therapies against solid tumors.

## Introduction

Adoptive T-cell therapy (ACT) has shown successful clinical outcomes against some cancer types. For example, chimeric antigen receptor-T (CAR-T) cells exhibited a high proportion of complete responses against B cell malignancies (1, 2) and multiple myeloma (3). T cell receptor-T (TCR-T) cells or tumor-infiltrating lymphocytes (TILs) have shown objective clinical response in synovial carcinoma (4) and melanoma (5), although the longevity of their therapeutic effect was limited. However, the majority of patients with solid tumors do not benefit from these therapies, likely owing to the impaired function of T cells in the suppressive tumor microenvironment (TME) (6, 7). This suggests that modulation of immune responses within the TME is crucial for improving the efficacy of ACTs against solid tumors.

Co-delivery of cytokines in conjunction with conventional ACT has proven to be an attractive approach (8), since it can both directly enhance the activity of transferred T cells (9–12) and modulate inhibitory immune cells in the suppressive TME (13, 14). The cytokine, interleukin-12 (IL-12), which is mainly produced by activated antigen presenting cells (15), has been extensively studied owing to its potent immune-activating and tumor-suppressive activities. In T cells, IL-12 signaling induces pro-inflammatory Th1 responses (16) while inhibiting the induction of regulatory T and Th17 cells (17, 18). IL-12 is also known to promote IFN-γ secretion and the cytotoxic potential of CD8 T and natural killer (NK) cells (19, 20). In response to IL-12, tumor cells can upregulate antigen presentation (21) and myeloid-derived suppressor cells can be converted to exhibit a T cell-supportive phenotype (22, 23). These observations have led to the engineering of T cells to secrete exogenous IL-12, which has been demonstrated to enhance the cytotoxic activity of T cells, deplete tumor-associated macrophages, and recruit innate immune cells to improve tumor control in animal models (13, 24–26). However, since the systemic exposure of IL-12 is poorly tolerated (27, 28), high serum levels of IL-12 released from engineered T cells have caused life-threatening side effects in clinical investigations (29). Thus, the safe exploitation of the therapeutic effect of IL-12 in ACT requires a novel approach that will allow the cytokine to be delivered locally at the tumor site in a tightly controlled manner.

When T cells are activated upon the recognition of antigens *via* the TCR, transcriptional and posttranslational regulation tightly coordinate the exact up- and downregulation of multiple genes (30) whose activity dysregulation may result in failure to control disease or the development of an autoimmune condition (31). Thus, the reprogramming of genes whose expression levels are induced by TCR signaling provides an attractive strategy for controlling transgene expression in a target cell-dependent manner. One such gene candidate is the immune checkpoint receptor, programmed cell death 1 (PD-1), which is well characterized for its critical inhibitory effects on T cells as well as its inducible expression upon TCR activation (32). The transient expression of PD-1 rapidly declines to basal levels in the absence of TCR signaling, which critically minimizes transgene expression outside of the tumor tissue. Furthermore, ablating inhibitory PD-1 expression on T cells alone has shown benefits in maintaining robust T cell activity (33, 34), suggesting that PD-1 may be an optimal target for the reprogrammed expression of exogenous IL-12.

Here, we aimed to rewrite the *PDCD1* locus to express IL-12 instead of PD-1 in New York esophageal squamous cell carcinoma 1 (NY-ESO-1)-specific T cells. To this end, we used clustered regularly interspaced short palindromic repeats (CRISPR)/CRISPR-associated protein 9 (Cas9) technology with recombinant adeno-associated virus 6 (AAV6) donors to knock-in a recombinant single-chain IL-12 sequence. The targeted insertion of IL-12 into the *PDCD1* locus resulted in the strict regulation of IL-12 expression through antigen-dependent T cell activation while simultaneously inactivating the expression of endogenous PD-1. The secretion of IL-12 from the *PDCD1* locus enhanced the effector function of NY-ESO-1 TCR-T cells and promoted their proliferation during repetitive tumor challenges *in vitro*, leading to superior anti-tumor activity in xenograft models.

## Materials and methods

### Cell lines

The A375 cell line was purchased from the American Type Culture Collection (ATCC). A375 cells were genetically engineered with lentivirus to generate Zsgreen-2a-Luciferase- or PD-L1-overexpressed A375 (A375-ZF or A375-PDL1) cells. The Lenti-X™ 293T and AAVpro^®^ 293T cell lines were purchased from Takara Bio. All cell lines were cultured in Dulbecco’s modified Eagle’s medium (DMEM; Welgene) supplemented with 10% heat-inactivated fetal bovine serum (Opti-Gold; Genedepot) and 1% penicillin/streptomycin (Gibco).

### Lentivirus production and transduction

To produce lentiviruses, Lenti-X™ 293T cells were transiently transfected with a lentiviral backbone and three packaging plasmids: pMD2.G (#12259; Addgene), pMDLg/pRRE (#12251; Addgene), and pRSV-Rev (#12253; Addgene). Briefly, 10 μg of each DNA was mixed with 120 μg of PEI MAX (Polysciences) in Opti-MEM (Gibco) and incubated at 37°C for 10 min. The mixture was added dropwise into HEK293T cells that had been plated onto 150-mm^2^ dishes 24 h before. After 6 h, the medium was replaced with fresh DMEM and the cells were maintained for 48 h. The culture supernatants containing lentivirus were collected and filtered using a 0.45-μm polyethersulfone membrane filter. Unpurified viral supernatants were used for the transduction of cell lines. For the transduction of human primary T cells, viral supernatants were further purified by ultracentrifugation. The viral supernatants were overlaid on 10% sucrose in Dulbecco’s phosphate-buffered saline (DPBS) and then ultracentrifuged at 25,000 rpm for 2 h at 4°C. After centrifugation, the cleared supernatants were removed and DBPS was added to the lentivirus pellet without resuspension. After overnight incubation at 4°C, the virus was resuspended and stored at –80°C. To transduce the cells with lentivirus, 1 × 10^6^ cells were mixed with purified or unpurified viruses in culture medium containing 30 μg of protamine sulfate (Sigma-Aldrich). Spin inoculation was performed by centrifugation at 1,000 × *g* for 90 min at 32°C, and thereafter the cells were maintained at 37°C. After 24 h, the transduced cell medium was replaced with fresh culture medium.

### AAV vector construction and production

The gene encoding human single-chain IL-12 (scIL-12; p40 and p35 subunits connected with the G6S linker), which was adopted from a previous report (25), was synthesized by Integrated DNA Technologies and cloned into the AAV-backbone plasmid (#20296; Addgene). The promoterless donor sequence encoded the knock-in donor genes flanked by two homology arms (614-bp left homology arm and 658-bp right homology arm), a self-cleaving T2A in-frame with a guide RNA cut site, followed by scIL-12, a self-cleaving P2A, truncated low-affinity nerve growth factor (tLNGFR), and the bovine growth hormone polyA signal (bGHpA).

For the production of recombinant AAV-6 virus, AAVpro^®^ 293T cells were transiently transfected with an AAV backbone plasmid and two packaging plasmids (pHelper and pRC-6; #6665; Clontech). Briefly, a mixture of DNA and PEI MAX was transfected as described above for lentivirus production. After 6 h, the medium was replaced with fresh culture medium and the cells were maintained for 72 h. AAV was purified from both the culture supernatant and pelleted cells by iodixanol-based density gradient ultracentrifugation, as previously described (35). The titers of recombinant AAV6 were determined by quantitative PCR using inverted terminal repeat-targeting primers (36).

### Single-guide RNA and Cas9 protein

The sequence of the guide RNA targeting exon 1 of the *PDCD1* locus (5′- GGCCAGGATGGTTCTTAGGT-3′) was designed using the web-based guide RNA design platform, CRISPR RGEN Tools (http://www.rgenome.net/). The single-guide RNA (sgRNA) was transcribed *in vitro* and purified as previously described (37). Immediately before electroporation, Cas9 protein (Enzynomics) and PDCD1 sgRNA were mixed at a 1:5 molar ratio and incubated for 10 min at 37°C to prepare the PD-1 targeting ribonucleoprotein (RNP).

### Genetic engineering of human primary T cells

The blood of an anonymous healthy human donor was acquired from ASAN Medical Center (Seoul, Korea) under a protocol approved by the institutional review board. Peripheral blood mononuclear cells (PBMCs) were isolated from the whole blood by density gradient centrifugation using Sepmate-50 Tubes (STEMCELL Technologies) and cryopreserved in freezing medium (90% FBS/10% dimethylsulfoxide) until use. Frozen PBMCs were thawed and CD3+ human primary T cells were purified using a Pan T-cell isolation kit (Miltenyi Biotec). The resulting T cells were activated using Dynabeads Human T-Activator CD3/CD28 (Thermo Fisher Scientific) at a 1:1 bead-to-cell ratio. One day after activation, T cells were collected and transduced with a lentivirus encoding NY-ESO-1-specific TCR. After overnight incubation, the culture medium of the TCR-transduced T cells was replaced with fresh culture medium. Two days after transduction, the T cells were collected and the Dynabeads were magnetically removed to perform genome editing. A Neon Transfection System 10 μL Kit (Invitrogen) was used for the electroporation of CRISPR RNP. First, 1 × 10^6^ NY-ESO-1 TCR-transduced T cells were resuspended in T buffer, mixed with PD-1-targeting RNP, and electroporated (1,400 V, 10 ms, 3 pulses). Electroporated T cells were transferred to fresh culture medium and maintained at 37°C for 15 min. The T cells were diluted to 0.5 × 10^6^ cells mL^–1^ with culture medium, and recombinant AAV6 virus was added at a multiplicity of infection of 5 × 10^4^. After 24 h, the culture medium was replaced with fresh culture medium with a cell density of 0.5 × 10^6^ cells mL^–1^. The medium was changed every 2 days. T cells were cultured in a T cell medium consisting of RPMI1640 (Gibco), 10% FBS (Gibco), 2 mM GlutaMAX (Gibco), 1 mM sodium pyruvate (Gibco), 55 μM 2-mercaptoethanol (Thermo Fisher Scientific), 10 mM HEPES (Sigma-Aldrich), and 1% non-essential amino acids (Gibco), supplemented with recombinant human interleukin-2 (300 IU mL^–1^; BMI Korea).

### Stimulation for detection of PD-1 and tLNGFR upregulation

To investigate activation-dependent transgene upregulation, 1 × 10^6^ NY-ESO-1 TCR-T cells were stimulated with 5 μg of plate-coated CD3 antibody (clone, OKT3; Bio X Cell) and 2 μg of soluble CD28 antibody (clone, CD28.2; Bio X Cell). After 48 h, stimulated T cells were collected and PD-1 upregulation and tLNGFR expression were analyzed using flow cytometry.

### Flow cytometry

For the detection of cell surface marker, 2 × 10^5^ T cells were washed and probed with antibodies in FACS buffer (1% bovine serum albumin [BSA] in DPBS) for 20 min at 4°C. To exclude the dead cell population, cells were stained with the fixable vitality dye, eFluor 780 (65-0865-14; eBioscience) for 10 min at 25°C. After being washed with FACS buffer, the cells were probed with chloroform-conjugated specific antibodies. NY-ESO-1-targeting TCR expression was determined with allophycocyanin (APC)-conjugated TCR Vβ13.1 antibody (362410; BioLegend), and CD3ϵ and TCR α/β were detected with Alexa Fluor^®^ 488 anti-human TCR α/β antibody (306712; BioLegend) and Brilliant Violet (BV)-421-conjugated CD3 antibody (300434; BioLegend). PD-1 upregulation was analyzed using BV421-conjugated CD279 antibody (367422; BioLegend). tLNGFR expression was analyzed using APC-conjugated CD271 antibody (130-113-418; Miltenyi Biotec). To detect CD4 and CD8, PerCP-CP/Cyanine5.5-conjugated CD4 antibody (357414; BioLegend) and APC-conjugated CD8 antibody (344722; BioLegend) were used. To determine differentiation status, BV421-conjugated CD45RO antibody (562641; BD Biosciences) and PE-conjugated CD197 antibody (560765; BD Biosciences) were used. To analyze intracellular proteins, surface-stained cells were fixed and permeabilized using a Cytofix/Cytoperm Fixation/Permeabilization Solution Kit (BD Biosciences). After being washed with intracellular staining buffer (1% BSA, 0.1% sodium azide, and 0.1% saponin in DPBS), the cells were probed with following antibodies. IFN-γ secretion was detected with BV711-conjugated IFN- γ antibody (564039; BD Bioscience). The releases of granzyme B (GzmB) and perforin were detected using APC-conjugated GzmB antibody (396408; BioLegend) and PE-conjugated perforin antibody (353304; BioLegend), respectively. The percentage of proliferative cells after repeated stimulation was analyzed with APC-conjugated Ki67 antibody (556027; BD Biosciences). All flow cytometry data were acquired with a BD LSRFortessa X-20 Cell Analyzer (BD Biosciences) and analyzed using the FlowJo software (BD Biosciences).

### Cytokine measurement

To detect IL-12 secretion after target-cell recognition, 2 × 10^5^ A375 cells were plated on 24-well tissue culture plates. After 20–24 h, 5 × 10^5^ NY-ESO-1 TCR-T cells resuspended in IL-2-free T cell culture medium were added to the A375 cells. After 2 days, the culture supernatant was collected and the secretion of IL-12 was measured using flow cytometry with a Human IL-12p70 Flex Set (BD Biosciences). To detect the releases of IFN-γ, TNF-α, IL-10, and IL-2, NY-ESO-1 TCR-T cells were co-cultured with A375 cells as described above and the cytokines released to the culture supernatant were measured using a cytometric bead array (CBA) assay with a Human th1/th2 Cytokine Kit (BD Biosciences).

### Western blotting

For the detection of STAT-4 and phospho-STAT4 (p-STAT4), 2 × 10^6^ NY-ESO-1 TCR-T cells were stimulated with 5 μg of plate-coated CD3 antibody for 72 h. To detect Bcl-xL upregulation, 2 × 10^5^ A375 cells were plated on 24-well tissue culture plates for 24 h, after which 1 × 10^6^ NY-ESO-1 TCR-T cells were added and maintained for 72 h. After stimulation, T cells were collected and lysed using NP-40 protein extraction buffer (Elpis Biotech) supplemented with a proteinase inhibitor cocktail (Sigma-Aldrich) and phosphatase inhibitor (Roche). The amount of protein in the lysates was quantified using a BCA protein assay kit (Thermo Fisher Scientific). Protein lysates (20 μg) from each sample were separated on precast 4%–12% Bis-Tris gradient gels (Invitrogen) using sodium dodecyl–sulfate polyacrylamide gel electrophoresis. Separated proteins were transferred to polyvinylidene fluoride (PVDF) membranes (Thermo Fisher Scientific) using an iBlot 2 Dry Blotting System (Thermo Fisher Scientific). Each membrane was blocked with 4% BSA in TBS with 0.5% Tween-20 (TBS-T) and probed with primary antibodies at 4°C overnight. The membranes were washed with TBS-T and incubated with secondary antibodies conjugated with horseradish peroxidase (HRP) at RT for 1 h. The following primary and secondary antibodies were used: anti-STAT4 (#2653, 1:1000; Cell Signaling), anti-phospho STAT4 (Tyr693) (#5267, 1:1000; Cell Signaling), anti-Bcl-xL (A19703, 1:1000; Abclonal, Wuhan, China), anti-actin (A2228, 1:20000; Sigma-Aldrich), anti-mouse IgG-HRP (#31430, 1:10000; Invitrogen), and anti-rabbit IgG-HRP (#31460, 1:10000; Invitrogen). Blot images were acquired using a ChemiDoc MP system (Bio-Rad) and processed using Image Lab software (Bio-Rad).

### Cytotoxicity assay

First, 2 × 10^4^ Zsgreen positive A375 cells were resuspended in 100 μL of culture medium and plated in 96-well tissue culture plates for 24 h. Then, 2 × 10^4^ NY-ESO-1 TCR-T cells were resuspended in 100 μL of the culture medium, added into A375 cells, and maintained for 120 h. The green signal from A375 cells was monitored every 2 h using an IncuCyte S3 Live-Cell Analysis System (Sartorius).

### Repeated tumor challenge

2 × 10^5^ PD-L1-overexpressed A375 cells were plated on culture plates and incubated for 24 h. The culture medium were replaced with fresh culture medium containing 10 μg mL^-1^ mitomycin C (Sigma-Aldrich). After incubation at 37 °C for 3h, A375 cells were washed with DPBS three times. For co-culture, 1 × 10^6^ NY-ESO-1 TCR-T cells were added into mitomycin C treated A375 cells. Four days later, T cells were counted using a Countess II Automated Cell Counter (Thermo Fisher Scientific) and re-challenged with fresh mitomycin C-pretreated A375-PDL1 cells. Three stimulations and cell counts were performed at intervals of four days. When the cells were counted, Trypan Blue (Gibco) was used to discriminate dead cells.

### Xenograft mouse model

Animal care and experiments were performed according to a protocol approved by the Animal Care Committee of the Korea Advanced Institute of Science and Technology. First, 1 × 10^6^ A375-ZF or A375-ZF-PDL1 cells were subcutaneously injected into the right flanks of 8–10-week-old male NSG mice (Jackson Laboratory). Mice were intravenously injected with 1 × 10^6^ NY-ESO-1^+^ TCR-T cells at 7 days after tumor injection in the A375-ZF model or at 6 days after tumor injection in the A375-ZF-PDL1 model. Tumor growth was monitored weekly using an IVIS^®^ Lumina II *In Vivo* Imaging System (PerkinElmer). Quantification of the luminescent signal was performed using the Living Image software (PerkinElmer). To investigate the infiltration of T cells at tumor sites, A375-ZF-PDL1-engrafted NSG mice were euthanized at 6 days after T cell injection, and tumors were harvested. The collected tumors were roughly chopped into small fragments (2–4 mm) and incubated with 20 μg of DNase I (Sigma-Aldrich) and 125 μg of collagenase IV (Sigma-Aldrich) with gentle shaking for 1 h at 37°C. After being washed with DPBS, the cells were treated with ACK Lysing Buffer (Gibco) and filtered through a 70-μm nylon mesh filter. The resulting single-cell suspension was analyzed by flow cytometry.

### Statistical analysis

All graph generations and statistical analyses were conducted using GraphPad Prism (GraphPad Software). Statistical significance was determined using two-tailed paired or unpaired Student’s *t*-test, one-way analysis of variance (ANOVA) with Tukey’s multiple comparisons, or two-way repeated-measures ANOVA with Holm-Sidak’s multiple comparisons test. For all analyses, a *P*-value <0.05 was considered statistically significant (*P < 0.05, **P < 0.01, ***P < 0.001, ****P < 0.0001).

## Results

### ΔPD-1-IL-12-edited NY-ESO-1-specific T cells secrete IL-12 in an antigen-dependent manner

**Figure 1:**
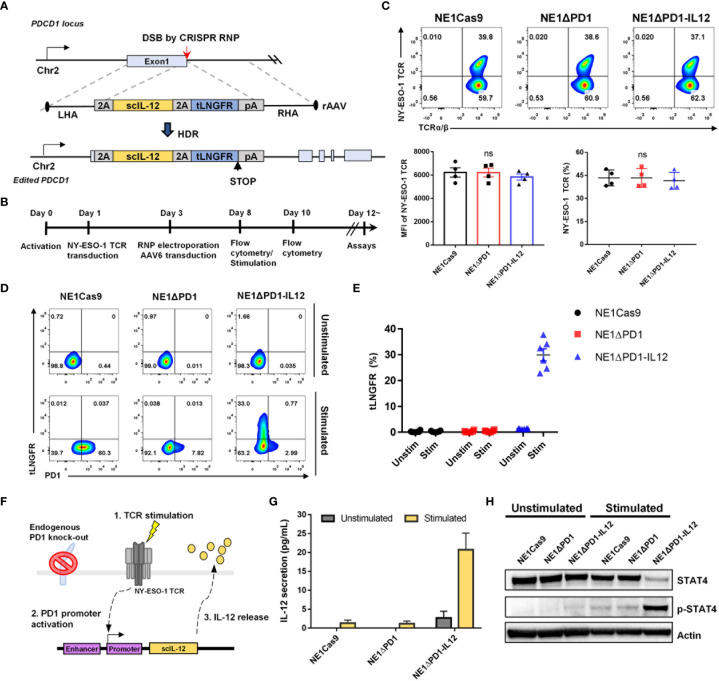
CRISPR-mediated *PDCD1* locus editing to generate ΔPD-1-IL-12-NY-ESO-1-specific T cells. **(A)** Schematic diagram for the targeted insertion of IL-12 into the PDCD1 locus using CRISPR RNP and AAV6 delivery (LHA and RHA: left and right homology arms, respectively, 2A: self-cleaving peptide, 2tLNGFR: truncated low-affinity nerve growth factor receptor, pA: polyA signal). **(B)** Timeline for the consecutive lentiviral transduction, electroporation, and AAV6 transduction to generate ΔPD-1-IL-12-edited NY-ESO-1-engineered T cells. **(C)** Five days after electroporation, the surface expression levels of NY-ESO-1 TCR on engineered T cells were analyzed using flow cytometry and the percentage and mean fluorescent intensity (MFI) of NY-ESO-1 TCR were quantified (n = 4; four independent experiments with four donors). Data were analyzed using one-way ANOVA. ns, not significant. **(D)** Representative flow cytometry plot of PD-1 and tLNGFR upregulation in engineered T cells two days after stimulation with αCD3 and αCD28. **(E)** Percentage of tLNGFR in engineered T cells before and after stimulation. Data are presented as the mean ± SEM (n = 6; six independent donors). **(F)** Schematic diagram of the stimulation-dependent release of targeted IL-12 from the edited PDCD1 locus. **(G)** After T cells were stimulated with A375 cells for 2 days, the amount of IL-12 secreted into the culture supernatant was measured using a CBA assay. Data are presented as the mean ± SEM (n = 3; three independent experiments with three donors). **(H)** Representative western blot image showing Tyr 693 phosphorylation of STAT-4 in NYESO1-ΔPD-1-IL-12 T cells before and after stimulation with αCD3 for 3 days (n = 3; three donors).

To insert the *IL-12* transgene into the *PDCD1* locus, we used CRISPR/Cas9 and AAV6-based knock-in systems, which have previously demonstrated robust and precise gene modifications in human T cells (38). To disrupt the *PDCD1* locus, we designed four sgRNAs targeting the first exon of *PDCD1* (**Supplementary Figure 1A**). We selected sgRNA#4 for further experiments because it resulted in a high knock-out efficiency (91.17%) when Cas9/sgRNA ribonucleoprotein (RNP) complexes were electroporated into activated human T cells (**Supplementary Figure 1B**). A promoterless AAV-6 donor matrix was designed to replace the endogenous PD-1 sequence with a single-chain IL-12 sequence, thus resulting in the expression of IL-12 under the control of *PDCD1* regulatory elements with concurrent knock-out of PD-1 expression. A self-cleaving P2A sequence was linked to the N-terminus of IL-12, followed by sequences encoding a self-cleaving T2A and truncated low-affinity nerve growth factor, tLNGFR, which was used as a surface marker to determine the knock-in efficiency (**Figure 1A**).

To edit the *PDCD1* locus of tumor-specific TCR-T cells that recognize the cancer testis antigen, NY-ESO-1_157–165_ SLLMWITQV (NY-ESO-1 TCR-T cells) (39), we first transduced T cells with lentivirus encoding NY-ESO-1 TCR and then subsequently conducted Cas9 RNP/AAV6 knock-in into transduced T cells (**Figure 1B**). This process did not influence the viability or expansion of the resulting ΔPD-1-IL-12 edited NY-ESO-1-specific T cells (NE1ΔPD-1-IL-12) (**Supplementary Figure 2**). As a control group, NY-ESO-1 TCR-T cells treated only with Cas9 (NE1Cas9) or NY-ESO-1 TCR-T cells treated with Cas9/sgRNA RNP without AAV (NE1ΔPD-1) were generated. Five days after electroporation and AAV donor transduction, the surface expression of NY-ESO-1 TCR was measured in each group using flow cytometry (**Figure 1C**). We confirmed that CRISPR editing after lentiviral transduction did not affect the expression of the transduced NY-ESO-1 TCR, as there was no significant difference between the three groups in the positive percentage or mean fluorescence intensity (MFI) of NY-ESO-1 TCR.

Next, we examined whether CRISPR knock-out or knock-in was successfully achieved by performing flow cytometry on day 10 (**Figure 1D**). Before stimulation, neither PD-1 nor tLNGFR was expressed in any group. Upon stimulation with anti-CD3 and anti-CD28 antibodies, about 60% of NE1Cas9 T cells became PD-1 positive, whereas less than 10% of NE1ΔPD-1 or NE1ΔPD-1-IL-12 T cells expressed PD-1. Additionally, up to 30% of NE1ΔPD-1-IL-12 T cells displayed tLNGFR expression after stimulation (**Figure 1E**), indicating that the donor sequence was successfully inserted in frame and expressed under the endogenous *PDCD1* regulatory elements upon T cell activation. Flawless integration of the transgene was also confirmed by in-out PCR analysis of genomic DNA from NE1ΔPD-1-IL-12 T cells (**Supplementary Figure 3**).

After confirming the accurate insertion of transgenes, we investigated whether IL-12 could be secreted in a target cell-dependent manner (**Figure 1F**). Engineered NY-ESO-1 TCR-T cells were co-cultured with NY-ESO-1^+^ A375 tumor cells for 48 h, and the amount of IL-12 secreted into the culture supernatants was analyzed using a CBA assay (**Figure 1G**). NE1Cas9 and NE1ΔPD-1 T cells did not produce IL-12 before co-culture, and NE1ΔPD-1-IL-12 T cells showed only slight leakage of IL-12 (2.662 pg/mL). Upon target cell recognition, NE1ΔPD-1-IL-12 T cells released approximately 20 pg/mL of IL-12 into the culture supernatant, whereas NE1Cas9 and NE1ΔPD-1 T cells showed minimal IL-12 release.

Given that *STAT4* is known as an early target gene of IL-12 signaling (40), we next examined the status of p-STAT4 in engineered T cells. Engineered NY-ESO-1 TCR-T cells were stimulated on anti-CD3-coated plates for 3 days and the levels of STAT4 and p-STAT4 were determined by western blotting (**Figure 1H**). Before stimulation, STAT-4 was not phosphorylated in any group. After stimulation, only NE1ΔPD-1-IL-12 T cells displayed a strong STAT-4 phosphorylation, which indicate the biological activity of the single-chain IL-12 released from the edited *PDCD1* locus. Overall, these results demonstrate that site-specific integration of the single-chain IL-12 gene into the *PDCD1* locus enabled functional IL-12 to be produced by NY-ESO-1-specific T cells in a target cell-dependent manner.

### The endogenous PD-1 promoter tightly regulates the knock-in transgene

**Figure 2:**
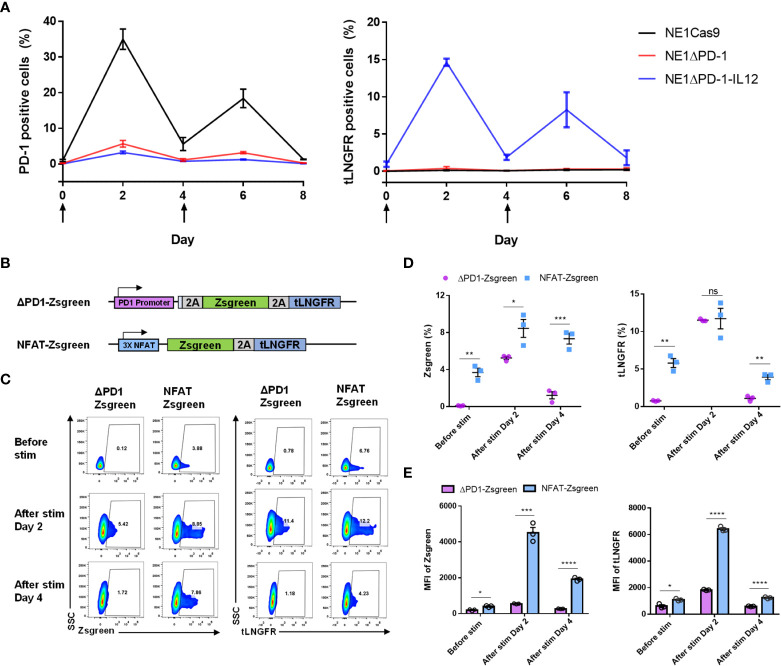
Tight control of transgene expression by the endogenous PDCD1 promoter. **(A)** Kinetics of PD-1 upregulation and tLNGFR expression in engineered T cells after two constitutive stimulations with αCD3 and αCD28. The arrow indicates the stimulation time points. Data are presented as the mean ± SEM (n = 3; independent experiments). **(B)** Schematic diagram of PD-1 promoter and NFAT-responsive promoter employed for expression of Zsgreen-2a-tLNGFR transgenes. **(C)** Representative flow cytometry plot for expression of Zsgreen and tLNGFR at 2-day intervals after stimulation with αCD3 and αCD28. **(D)** Percentage of Zsgreen and tLNGFR expression levels of engineered T cells in C. **(E)** MFI of Zsgreen and tLNGFR of engineered T cells in C. Data in D and E are presented as the mean ± SEM (n = 3; three independent experiments). P-values of D and E were determined by two-tailed unpaired t-test. *P<0.05, **P<0.01, ***P<0.001, and ****P<0.0001 were considered statistically significant. ns, not significant.

PD-1 expression is known to respond dynamically to TCR activation (41, 42). Here, we measured the expression levels of PD-1 and tLNGFR after stimulation of T cells to determine whether the transgenes inserted into the *PDCD1* locus would behave similarly (**Figure 2A**). The percentage of PD-1-positive cells in the NE1Cas9 T cells increased significantly at 2 days after stimulation, returned to almost baseline by day 4, and then re-elevated after the second stimulation. In contrast, minimal levels of PD-1-positive cells were detected in the NE1ΔPD-1 and NE1ΔPD-1-IL-12 T cell cells, confirming that the expression of inhibitory PD-1 was successfully abolished. Interestingly, the expression of tLNGFR was detected only in the NE1ΔPD-1-IL-12 T cells, where it showed kinetics similar to those of PD-1 expression in the NE1Cas9 T cells. These results indicate that our approach successfully utilizes the intrinsic regulatory mechanism of PD-1 to control transgene expression while simultaneously blocking the expression of endogenous PD-1.

As previously reported (25, 26), a synthetic promoter that responds to TCR activation could be used as an alternative strategy to our approach. Therefore, we next compared the expression patterns of a transgene inserted into the *PDCD1* locus with those controlled by a synthetic nuclear factor of activated T-cells (NFAT)-responsive promoter. To monitor expression, we used a transgene encoding a green fluorescent protein and tLNGFR (Zsgreen-2A-tLGNFR) which was either inserted site-specifically into the endogenous *PDCD1* locus using CRISPR knock-in (ΔPD-1-Zsgreen), or randomly integrated into the genome with the NFAT-responsive promoter using lentivirus (NFAT-Zsgreen, **Figure 2B**). After cells were stimulated with anti-CD3 and anti-CD28 antibodies, the upregulation of Zsgreen and tLNGFR was measured using flow cytometry (**Figure 2C**). Before stimulation, we found that NFAT-Zsgreen T cells exhibit leaky expression of Zsgreen and tLNGFR, which was not observed in ΔPD-1-Zsgreen T cells. After stimulation, consistent with the results obtained from NE1ΔPD-1-IL-12 T cells, ΔPD-1-Zsgreen T cells displayed tightly controlled transgene expression that was upregulated on day 2 and returned to the baseline by day 4. NFAT-Zsgreen T cells also showed inducible transgene up-regulation at 2 day post-stimulation, but to a significantly higher level than that seen for ΔPD-1-Zsgreen T cells, as determined by the percentages (**Figure 2D**) and MFI (**Figure 2E**) of Zsgreen+ and tLNGFR+ cells. Furthermore, unlike ΔPD-1-Zsgreen T cells, NFAT-Zsgreen T cells exhibited significant levels of residual transgene expression on day 4. The significant levels of leaky transgene in NFAT-Zsgreen T cells, together with their high expression levels upon stimulation, may account for the unexpected toxicity observed in patients infused with NFAT-driven IL-12-expressing T cells (29). Collectively, these results suggest that, compared to the NFAT-responsive promoter, PD-1 regulatory elements can provide a better control of transgene expression without leakage and induce moderate levels of transgene expression with rapid kinetics, which could mitigate the potential toxicity of IL-12 secreted by engineered T cells.

### NE1ΔPD-1-IL-12 T cells exhibit enhanced effector function *in vitro*

**Figure 3:**
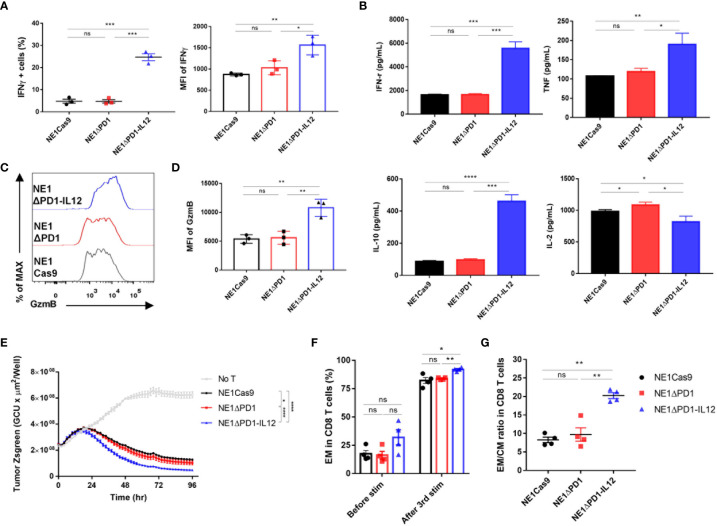
Enhanced effector function of ΔPD-1-IL-12-edited NY-ESO-1 TCR-T cells *in vitro* **(A)** Engineered T cells were co-cultured with A375 cells for 24 h. IFN-γ secretion was determined using flow cytometry. The percentage of IFN-γ positive cells and the MFI of IFN-γ were quantified. Data are presented as the mean ± SEM (n = 3; three independent experiments with three donors). **(B)** Engineered T cells were co-cultured with A375 cells for 48 h and the cytokines (IFN-γ, TNF, L-10, and IL-2) released into the culture supernatants were measured by CBA assay; results shown are representative of three experiments with three different donors. Data are presented as the mean ± SD of triplicates. **(C)** Engineered T cells were co-cultured with A375 cells for 48 h. The GzmB expression was determined by intracellular flow cytometric analysis. **(D)** The MFI of GzmB in the engineered T cells shown in **(C, E)** Engineered T cells were co-cultured with Zsgreen+ A375 cells for 96 h. The green signal from tumor cells was measured using IncuCyte every 2 h. A decrease in the green signal indicates that tumor cells were killed; results shown are representative of three experiments. Data are presented as the mean ± SD. **(F)** Percentage of effector memory CD8 T cells (CD45RO^+^/CCR7^–^) after a third repetitive stimulation with A375-PDL1 tumor cells. **(G)** The ratio of effector memory to central memory (CD45RO^+^/CCR7^+^) CD8 T cells in **(F)** Data of F and G are presented as the mean ± SEM (n = 4; individual donors). The *P*-values of A, B, and D were determined by two-tailed unpaired *t*-test. The *P*-value of E was determined by repeated-measures ANOVA followed by Holm-Sidak’s multiple comparisons test. Data of F and G were compared by two-tailed paired *t*-test. *P<0.05, **P<0.01, ***P<0.001, and ****P<0.0001 were considered statistically significant. ns, not significant.

Next, we investigated whether the IL-12 secreted from NE1ΔPD-1-IL-12 T cells could directly affect the effector function of TCR-T cells. As IL-12 signaling is known to be associated with increased IFN-γ production (19), we used intracellular flow cytometry to measure IFN-γ expression levels in engineered T cells co-cultured with A375 tumor cells for 24 h. Our results confirmed that NE1ΔPD-1-IL-12 T cells showed significantly higher proportions of IFN-γ-positive T cells and higher MFI compared to control T cells (**Figure 3A**). CBA-based analysis indicated that NE1ΔPD-1-IL-12 T cells produce higher levels of IFN-γ, TNF, and IL-10, but lower levels of IL-2, compared to control T cells (**Figure 3B**); this is consistent with the results from previous studies (13). NE1ΔPD-1-IL-12 T cells co-cultured with A375 tumor cells for 24 h also displayed higher expression of GzmB (**Figures 3C, D**), but no difference in the percentage or MFI for perforin, compared to control T cells (**Supplementary Figures 4A, B**). Next, we evaluated the cytotoxic function of the engineered TCR-T cells by co-culturing them with Zsgreen-overexpressing A375 cells. When the green signal on tumor cells was measured every 2 h by a live cell imaging system, NE1ΔPD-1-IL-12 T cells showed a more rapid decrease in the green signal compared to control T cells (**Figure 3E**). These results indicate that IL-12 secretion from the *PDCD1* locus enhances the effector function of NY-ESO-1-specific T cells, resulting in more efficient killing of target cancer cells.

IL-12 signaling has been reported to reprogram CD8+ T cells into effector memory and effector T cells (43, 44), which can elicit immediate effector functions in response to antigen recognition. Thus, we analyzed the differentiation status of the T cells before and after repeated exposure to target cells, based on the expression levels of CD45RO and C-C motif chemokine receptor 7 (CCR7) (**Supplementary Figures 5A, B**). Under homeostatic expansion conditions, the proportion of CD8+ T cells was similar among all three groups (**Supplementary Figure 5C**). After repeated stimulation, most T cells were skewed toward the CD8 phenotype, but the percentage remained similar in all groups. The proportion of effector memory CD8 T cells (CD45RO^+^CCR7^–^) was higher among NE1ΔPD-1-IL-12 T cells than in the control group (**Figure 3F**). The ratio of effector memory CD8 T cells to central memory CD8 T cells (CD45RO^+^CCR7^+^) was also increased in NE1ΔPD-1-IL-12 T cells (**Figure 3G**), indicating that *PDCD1*-driven*-*IL-12 secretion during the repetitive exposure to antigens had impacted the differentiation of CD8^+^ T cells. Similar changes in effector and central memory T cell pool were observed in CD4^+^ T cells (**Supplementary Figures 5B, D**).

### NE1ΔPD-1-IL-12 T cells greatly expand during chronic antigen stimulation

**Figure 4:**
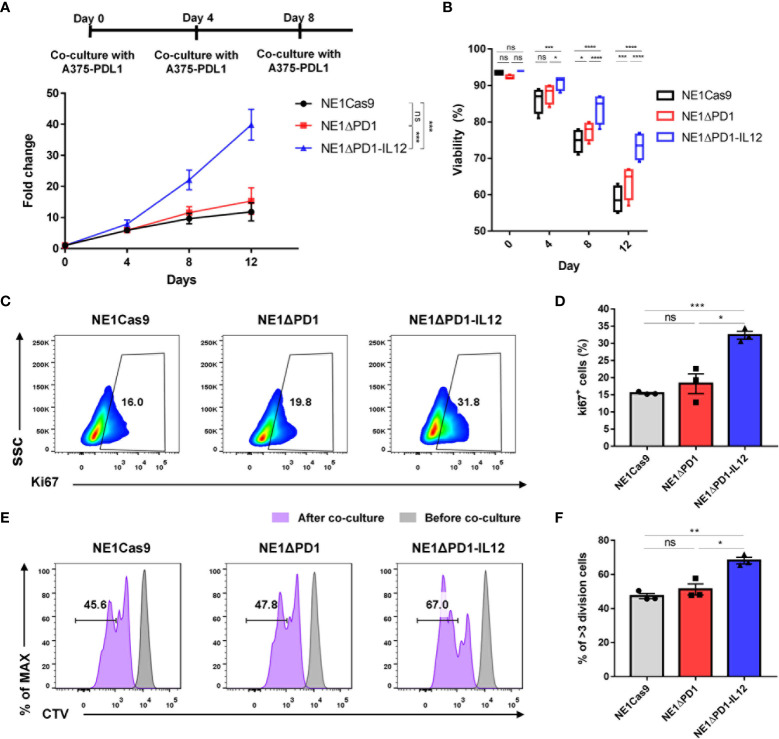
Enhanced cell expansion of ΔPD-1-IL-12-edited NY-ESO-1 TCR-T cells during repeated tumor challenge **(A)** The expansion engineered T cells was analyzed after repetitive stimulation with mitomycin C-pretreated A375-PDL1 cells three times every 4 d. Data are presented as the mean ± SEM (n = 4; four independent experiments with individual donors). **(B)** The viability of expanded T cells in A was measured using a Countess II automated cell counter with Trypan Blue staining. **(C)** Representative flow plot for Ki67 expression after the third stimulation in **(A, D)** The percentage of Ki67^+^ T cells after the third stimulation (n = 3; individual donors). **(E)** Cell trace violet (CV)-stained engineered T cells were co-cultured with A375-PDL1 for 3 days. The diluted CTV intensity was measured by flow cytometry. **(F)** Percentage of diluted CTV stained T cells that divided more than three times in E (n = 3; individual donors). All data are presented as the mean ± SEM. The *P*-values of A and B were determined by repeated-measures ANOVA followed by Holm-Sidak’s multiple comparisons test. *P*-values of D and F were determined by two-tailed unpaired *t*-test. *P<0.05, **P<0.01, ***P<0.001, and ****P<0.0001 were considered statistically significant. ns, not significant.

The immunosuppressive TME is believed to inhibit the proliferation and survival of adoptively transferred T cells (7, 45). Since proper T cell expansion is crucial for the success of T cell therapy (46, 47), we investigated the expansion ability during repeated tumor challenges. NY-ESO-1-specific T cells were repeatedly co-cultured with A375-PDL1 cells that had been pretreated with mitomycin C 3 times at 4-day intervals, and cell numbers and viability were monitored by cell counting with Trypan Blue staining (**Figure 4A**). After the first antigen stimulation, there was no difference in the number of cells among the three groups. However, after the second and third challenges, NE1ΔPD-1-IL-12 T cells were significantly more abundant than control T cells. Furthermore, although repeated stimulation gradually reduced the overall viability in all groups, NE1ΔPD-1-IL-12 T cells showed higher viability at all tested time points compared to control T cells (**Figure 4B**).

Next, we investigated whether this enhanced expansion of NE1ΔPD-1-IL-12 T cells was due to changes in apoptosis resistance or proliferative capacity. As a previous study demonstrated that IL-12 signaling inhibits TCR-induced T cell death by regulating caspases and anti-apoptotic molecules (48), we measured the percentage of annexin V-positive cells. However, we found no between-group difference after the third stimulation (**Supplementary Figure 5A**). We also found that the expression levels of the antiapoptotic marker, Bcl-xL, and the master anti-apoptotic regulator, c-FLIP, were similar in all groups (**Supplementary Figures 5B, C**). The levels of cleaved caspase-3 and -8 were also similar across all groups (**Supplementary Figures 5D, E**). These findings suggest that apoptosis resistance may not significantly contribute to the increased number of NE1ΔPD-1-IL-12 T cells observed following repeated stimulation. In contrast, when the proliferative capacity of T cells was evaluated by measuring Ki67 expression after the third stimulation, NE1ΔPD-1-IL-12 T cells showed a significantly higher percentage of Ki67 positive cells compared to control T cells (**Figures 4C, D**). Consistent with this, NE1ΔPD-1-IL-12 T cells co-cultured with A375-PDL1 cells for 3 days showed a more divided cell population than the other groups, as determined by cell-trace violet dye staining (**Figures 4E, F**). Taken together, our results indicate that IL-12 production from the edited *PDCD1* locus contributed to expanding NY-ESO-1-specific T cells under chronic antigen stimulation by enhancing their proliferative capacity.

### NE1ΔPD-1-IL-12 T cells exhibit superior anti-tumor activity *in vivo*

**Figure 5:**
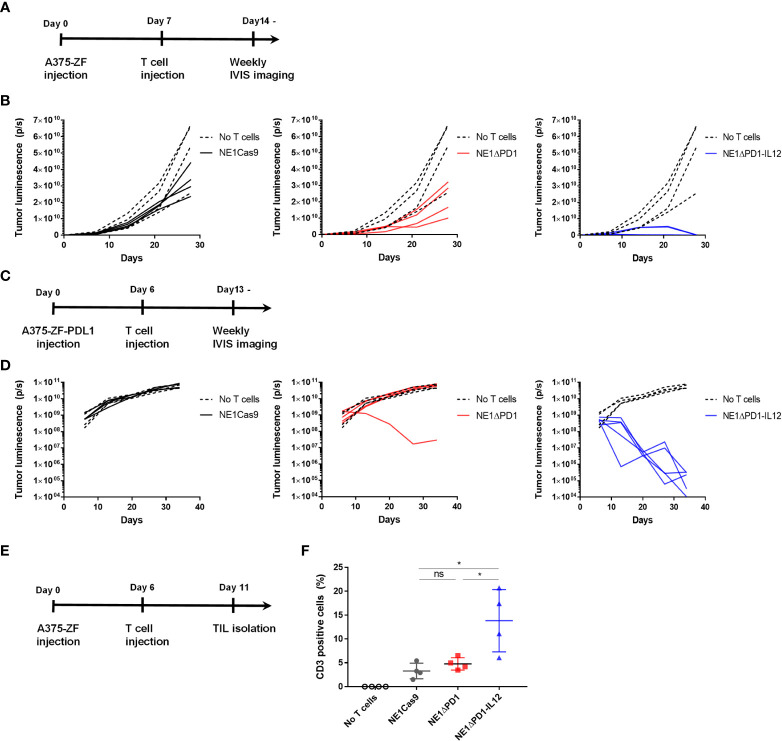
Enhanced anti-tumor activity of ΔPD-1-IL-12-edited NY-ESO-1 TCR-T cells *in vivo* **(A)** Timeline for *in vivo* experiment performed with A375-ZF tumor cells. Immunodeficient NSG mice were subcutaneously injected with 1 × 10^6^ A375-ZF cells. After 7 days, 1 × 10^6^ NY-ESO-1+ T cells were intravenously injected. The luminescence signal from tumor cells was measured weakly using an *in vivo* imaging system (IVIS). **(B)** Quantitative analysis of bioluminescent signal from individual mouse. ΔPD-1-IL-12 T cells exhibited superior anti-tumor activity (n = 4 mice per group). **(C)** Timeline for *in vivo* experiments performed using the PD-L1-overexpressed A375 model. Six days after subcutaneous injection of A375-ZF-PDL1 cells, 1 × 10^6^ NY-ESO-1^+^ T cells were intravenously injected. The luminescence signal from tumor cells was measured weekly using IVIS. **(D)** Quantitative analysis of bioluminescent signal from individual mouse. Tumors were cleared in only one of the PD-1 deleted NY-SO-1-treated mice. All NE1ΔPD-1-IL-12-treated mice were cured from tumors (n = 4 mice for no T cells and NE1Cas9, n = 5 mice for NE1ΔPD-1 and NE1ΔPD-1-IL-12). **(E)** Timeline for investigating engineered T cells infiltrated into the tumor site. A375-PDL1 tumor cells and NY-ESO-1 specific T cells were injected as in **(C)** At 11 d, the mice were sacrificed and tumors were harvested. Tumors were mechanically dissociated and the infiltrated T cells were analyzed by flow cytometry. **(F)** The percentage of CD3+ cells in tumor tissues (n = 4 mice per group). Data are presented as mean ± SEM. The *P*-value was determined by two-tailed unpaired *t*-test. *P<0.05 was considered statistically significant. ns, not significant.

To investigate the therapeutic efficacy of NE1ΔPD-1-IL-12 T cells *in vivo*, we subcutaneously implanted immune-deficient NSG mice with 1 × 10^6^ Zsgreen and firefly luciferase-overexpressing A375 cells (A375-ZF), followed by intravenous injection of 1 × 10^6^ NY-ESO-1^+^ TCR-T cells (**Figure 5A**). When the bioluminescent signal was measured weakly to monitor the tumor burden, the tumor cells were found to be completely eradicated in mice treated with NE1ΔPD-1-IL-12 T cells (**Figure 5B**, **Supplementary Figures 7A, B**). In contrast, NE1Cas9 and NE1ΔPD-1 T cells failed to control tumor growth in this xenograft model, suggesting that the secretion of IL-12 from TCR-T cells played a critical role in tumor control. We then carried out another *in vivo* experiment in which we used PD-L1-overexpressing A375 cells (A375-ZF-PDL1) to mimic a more immunosuppressive TME (**Figure 5C**). Under these experimental conditions, NE1Cas9 T cells had virtually no effect on tumor growth; NE1ΔPD-1 T cells cleared the tumor in one out of five mice, likely reflecting the effect of PD-1 knockout in the T cells; and NE1ΔPD-1-IL-12 T cells completely eradicated the tumors in all five mice, further demonstrating the potent anti-tumor activity of NE1ΔPD-1-IL-12 T cells (**Figure 5C**, **Supplementary Figure 7C**). Lastly, to determine the degree of T cell expansion *in vivo*, we analyzed isolated tumor tissues at 5 day after T cell injection (**Figure 5E**). The harvested tumors were mechanically dissociated and filtered to generate single-cell suspension and analyzed using flow cytometry (**Supplementary Figure 8**). We observed a significantly higher proportion of CD3^+^ T cells in the mice treated with ΔPD-1-IL-12 T cells compared to those of the control groups (**Figure 5F**). Overall, consistent with the findings from our *in vitro* experiments, the results from *in vivo* xenograft models confirmed that the engineering of T cells to secrete IL-12 from the *PDCD1* locus can profoundly enhance the anti-tumor activity of NY-ESO-1-specific TCR-T cells.

## Discussion

A variety of cofactors, including soluble factors such as cytokines or chemokines, as well as membrane-bound factors such as co-stimulatory receptors or cytokine receptors, have been employed to design ACTs with enhanced therapeutic potential (49–51). The co-delivery of exogenous cytokines has been extensively investigated because such factors have pleiotropic effects on both the therapeutic T cell itself and on other immune and non-immune cells of the TME (52). However, the constitutive secretion of some cytokines from circulating ACTs raises potential safety concerns, given that systemic administration of these cytokines often leads to severe side effects in patients due to on-target off-tumor toxicity (28, 53, 54). Therefore, a novel ACT engineering strategy that can limit their cytokine expression within tumor tissues could help maximize the therapeutic benefits while minimizing the potential adverse effects.

To address this goal, we employed an endogenous genetic network influenced by TCR signaling to antigen-dependently control the expression of IL-12, a potent pro-inflammatory cytokine that has long been investigated in cancer immunotherapy. Based on reports that the *PDCD1* gene shows tight and dynamically regulated expression in response to T cell activation (32, 42), we selected this locus for targeted insertion of the *IL-12* gene using the CRISPR/Cas9 genome editing tool. This strategy enabled us to express IL-12 under the control of *PDCD1* regulatory elements strictly in response to T cell activation, with expression kinetics similar to those of endogenous PD-1. In this manner, the engineered T cells were expected to secrete IL-12 locally only upon encountering antigens within the tumor tissue. In addition, CRISPR editing of the *PDCD1* enabled us to simultaneously knock out the PD-1 gene, further empowering T cells to resist the functional exhaustion caused by inhibitory PD-1 signaling (45). Indeed, several studies have demonstrated that the disruption of PD-1 using CRISPR results in the enhanced anti-tumor activity of CAR-T cells (33, 55, 56), which was also partially observed in our *in vitro* and *in vivo* experiments with NY-ESO-1 T cells lacking PD-1 expression (NE1ΔPD-1 T cells).

Our results demonstrated that insertion of the *IL-12* gene into the *PDCD1* locus induces a more moderate expression level of IL-12 with a more strict reliance on TCR activation compared to that driven by the NFAT-responsive promoter. In a previous study, genetically engineered TILs expressing NFAT-IL-12 exhibited unexpected toxicity in patients (29), which may be attributable to a high amount of released IL-12. This report further described that low levels of leaky constitutive expression of IL-12 from the engineered TILs exert anti-proliferative effect, leading to difficulties in growing sufficient numbers of cells *ex vivo* and likely contributing to the poor persistence of cells *in vivo*. Another clinical study (NCT02498912) is currently underway using CAR-T cells engineered to release IL-12 from an internal ribosome entry site (IRES) positioned immediately after the CAR sequence in the vector (57, 58). The authors of this study hypothesized that the release of less IL-12 from IRES-IL-12 (200 pg/mL) compared to NFAT-IL-12 (50,000 pg/mL) might minimize potential IL-12-related toxicity issues. Of note, our ΔPD-1-IL-12-edited T cells secreted even fewer IL-12 (20 pg/mL) than the T cells engineered with NFAT-IL-12 or IRES-IL-12, and thus might offer even more benefits for preventing the potential adverse effects associated with IL-12. Nevertheless, a more thorough analysis with respect to potential IL-12-related safety concerns must be conducted before implementing our approach in clinical settings.

It is important to note that the moderate levels of IL-12 produced in our system were sufficient to elicit superior anti-tumor activity from ΔPD-1-IL-12-edited NY-ESO-1 T cells compared with non-edited NY-ESO-1 T cells in both *in vitro* cytotoxicity assays and *in vivo* xenograft mouse models. The expression of IL-12 in our system was significantly lower (20 pg/ml) than in previously reported systems such as IRES-IL-12 (200 pg/ml) (57), TET-IL-12 (1000 pg/ml) (59), and NFAT-IL-12 (5000 pg/ml) (29). Different copy numbers of the transgene as well as different promoter kinetics and strength may account for this difference. Unlike retroviral vector systems used in previous approaches, which randomly integrate multiple copies of transgenes into the host genome, our CRISPR knock-in system allows us to add a single copy of transgene per chromosome precisely and to control its expression through the endogenous transcription machinery of the PDCD1 locus. Even with the low levels of IL-12 released by activated T cells, we observed along with the increased production of pro-inflammatory cytokines, such as IFN-γ and TNF, the enhanced expression of GzmB, which is a major effector molecule of T cells for inducing apoptosis in tumor cells. This appears to be linked to the superior anti-tumor activity of PD-1-IL-12 edited T cells. We also found that the transient expression of IL-12 significantly enhanced the expansion of ΔPD-1-IL-12-edited T cells by promoting their proliferation upon repeated antigen stimulation. This enhanced proliferation is in sharp contrast to a previous report, which showed that continuous retroviral IL-12 expression has deleterious effects on T cell proliferation (25). The proliferation enhancement found in our system may be particularly important for the enhanced anti-tumor activity observed *in vivo* for ΔPD-1-IL-12-edited T cells. Collectively, our results suggest that the target-dependent and moderate expression of IL-12 derived from the *PDCD1* locus provides an effective strategy to enhance the anti-tumor function of TCR-T cells.

Several previous studies have demonstrated that IL-12 is involved in regulating not only T cells, but also a wide range of other immune cells, such as DCs and macrophages (13, 15, 24, 26). However, the severe immune-compromised state of NSG mice and a lack of cross-reactivity between human IL-12 and mouse IL-12 receptor proteins precluded us from investigating this axis in our study. The humanized mice model, in which human CD34+ HSCs are engrafted into NSG mice, may allow us to study the effects of IL-12 released by ΔPD-1-IL-12-edited T cells on other immune cell types in a more comprehensive manner.

In summary, we herein demonstrate that the inducible genetic circuit of PD-1 expression could be reprogrammed to secrete IL-12 in NY-ESO-1 TCR-T cells using CRISPR knock-in technology. The modest and tight expression of IL-12 from the *PDCD1* locus was sufficient to enhance the anti-tumor activity of NY-ESO-1 TCR-T cells. Our strategy could be extended to the controlled expression of other proteins of interest, such as antibodies, cytokines, chemokines, receptors, and transcription factors that may enhance or synergize with the function of T cells. Other genetic loci in addition to the *PDCD1* locus could also be explored in future studies. Lastly, our approach may provide a novel engineering approach for other adoptive T cell therapies, such as CAR-T, TIL, and virus-specific T cell therapies, against solid tumors.

## Data availability statement

The original contributions presented in the study are included in the article/**Supplementary Material**. Further inquiries can be directed to the corresponding author.

## Ethics statement

The animal study was reviewed and approved by Animal Care Committee of the Korea Advanced Institute of Science and Technology.

## Author contributions

SK designed the study, performed the experiments, analyzed the data, and wrote the manuscript. CIP assisted in the *in vitro* and *in vivo* experiments. SL and HRC assisted in the *in vivo* experiments. CHK supervised the study and wrote the manuscript. All authors contributed to the article and approved the submitted version.
