# Supplementary material for: Reprogramming of IL-12 secretion in the PDCD1 locus improves the anti-tumor activity of NY-ESO-1 TCR-T cells
Source: Front Immunol. 2023 Jan 30;14:1062365. doi: 10.3389/fimmu.2023.1062365 (PMC9923015; doi:10.3389/fimmu.2023.1062365)
Supplement: Supplementary file 1 [file Presentation_1.pptx]

## Slide 1
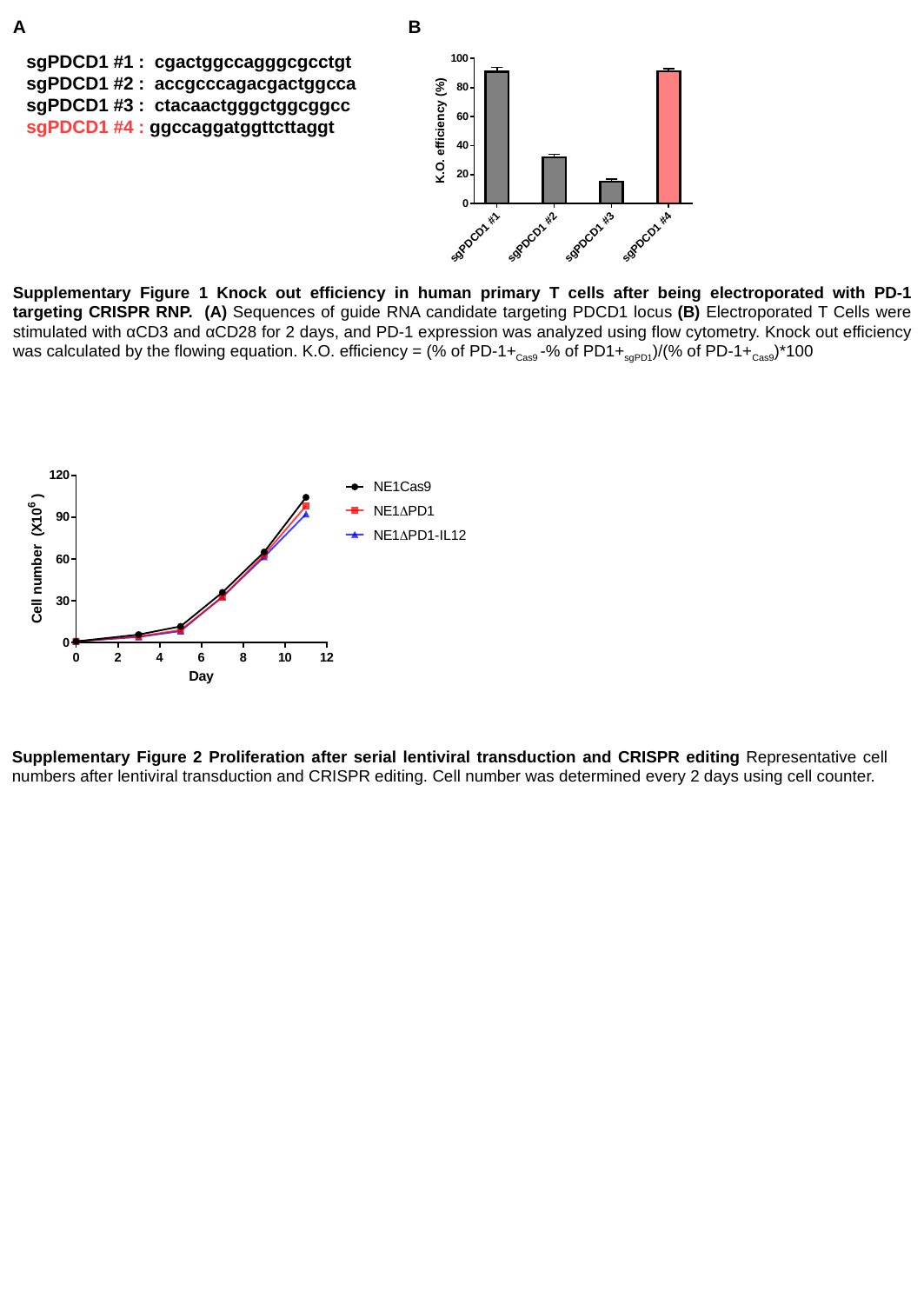

A
B
sgPDCD1 #1 : cgactggccagggcgcctgt
sgPDCD1 #2 : accgcccagacgactggcca
sgPDCD1 #3 : ctacaactgggctggcggcc
sgPDCD1 #4 : ggccaggatggttcttaggt
Supplementary Figure 1 Knock out efficiency in human primary T cells after being electroporated with PD-1 targeting CRISPR RNP. (A) Sequences of guide RNA candidate targeting PDCD1 locus (B) Electroporated T Cells were stimulated with αCD3 and αCD28 for 2 days, and PD-1 expression was analyzed using flow cytometry. Knock out efficiency was calculated by the flowing equation. K.O. efficiency = (% of PD-1+Cas9 -% of PD1+sgPD1)/(% of PD-1+Cas9)*100
Supplementary Figure 2 Proliferation after serial lentiviral transduction and CRISPR editing Representative cell numbers after lentiviral transduction and CRISPR editing. Cell number was determined every 2 days using cell counter.

## Slide 2
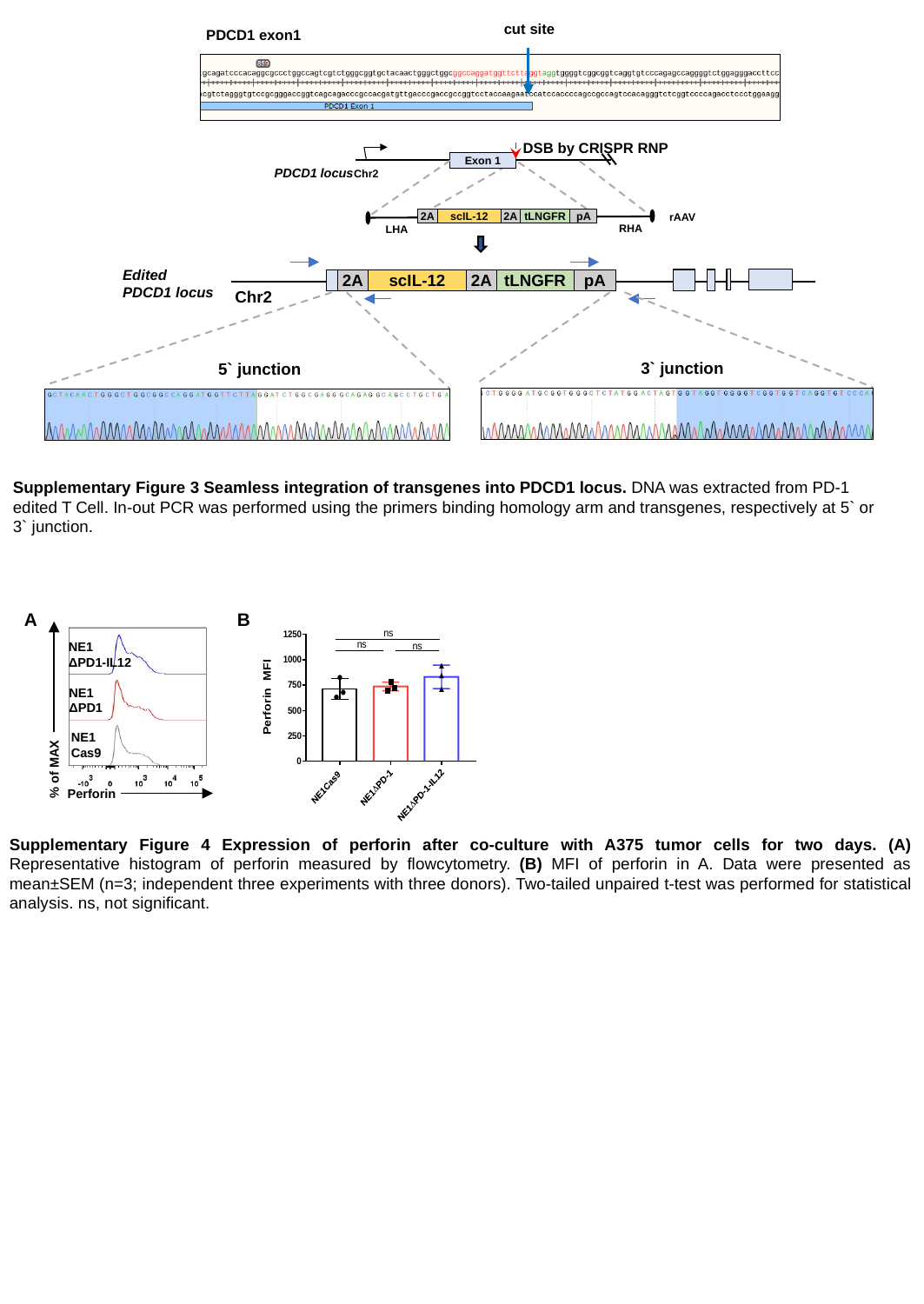

cut site
PDCD1 exon1
DSB by CRISPR RNP
Exon 1
PDCD1 locus
Chr2
rAAV
2A
pA
tLNGFR
2A
scIL-12
RHA
LHA
Edited
PDCD1 locus
2A
pA
2A
scIL-12
tLNGFR
Chr2
3` junction
5` junction
Supplementary Figure 3 Seamless integration of transgenes into PDCD1 locus. DNA was extracted from PD-1 edited T Cell. In-out PCR was performed using the primers binding homology arm and transgenes, respectively at 5` or 3` junction.
A
B
NE1
ΔPD1-IL12
NE1
ΔPD1
NE1
Cas9
Perforin
% of MAX
Supplementary Figure 4 Expression of perforin after co-culture with A375 tumor cells for two days. (A) Representative histogram of perforin measured by flowcytometry. (B) MFI of perforin in A. Data were presented as mean±SEM (n=3; independent three experiments with three donors). Two-tailed unpaired t-test was performed for statistical analysis. ns, not significant.

## Slide 3
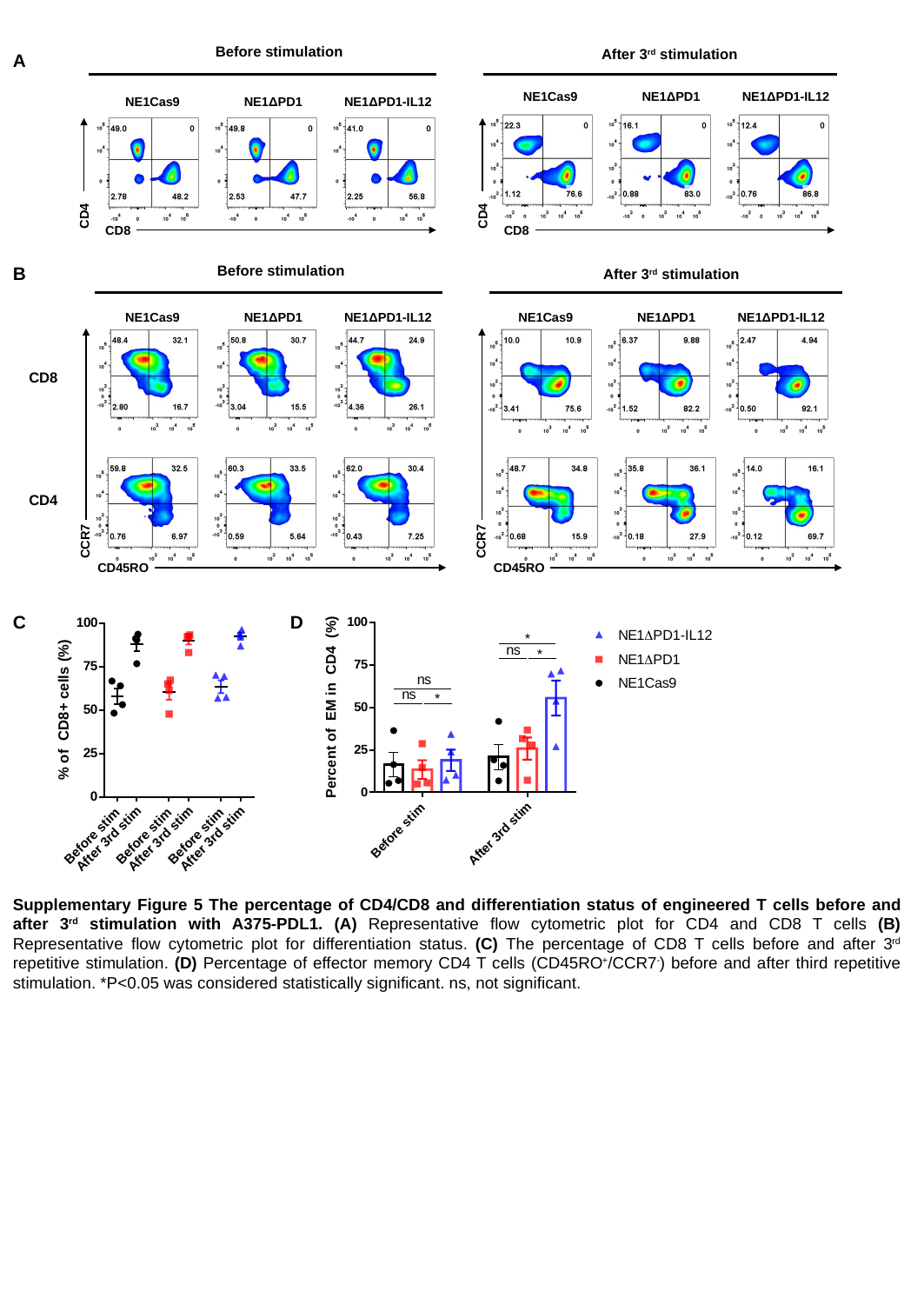

Before stimulation
After 3rd stimulation
NE1Cas9
NE1ΔPD1
NE1ΔPD1-IL12
CD4
CD8
CD4
CD8
CCR7
CD45RO
CCR7
CD45RO
CD8
CD4
A
NE1Cas9
NE1ΔPD1
NE1ΔPD1-IL12
B
Before stimulation
After 3rd stimulation
NE1Cas9
NE1ΔPD1
NE1ΔPD1-IL12
NE1Cas9
NE1ΔPD1
NE1ΔPD1-IL12
C
D
Supplementary Figure 5 The percentage of CD4/CD8 and differentiation status of engineered T cells before and after 3rd stimulation with A375-PDL1. (A) Representative flow cytometric plot for CD4 and CD8 T cells (B) Representative flow cytometric plot for differentiation status. (C) The percentage of CD8 T cells before and after 3rd repetitive stimulation. (D) Percentage of effector memory CD4 T cells (CD45RO+/CCR7-) before and after third repetitive stimulation. *P<0.05 was considered statistically significant. ns, not significant.

## Slide 4
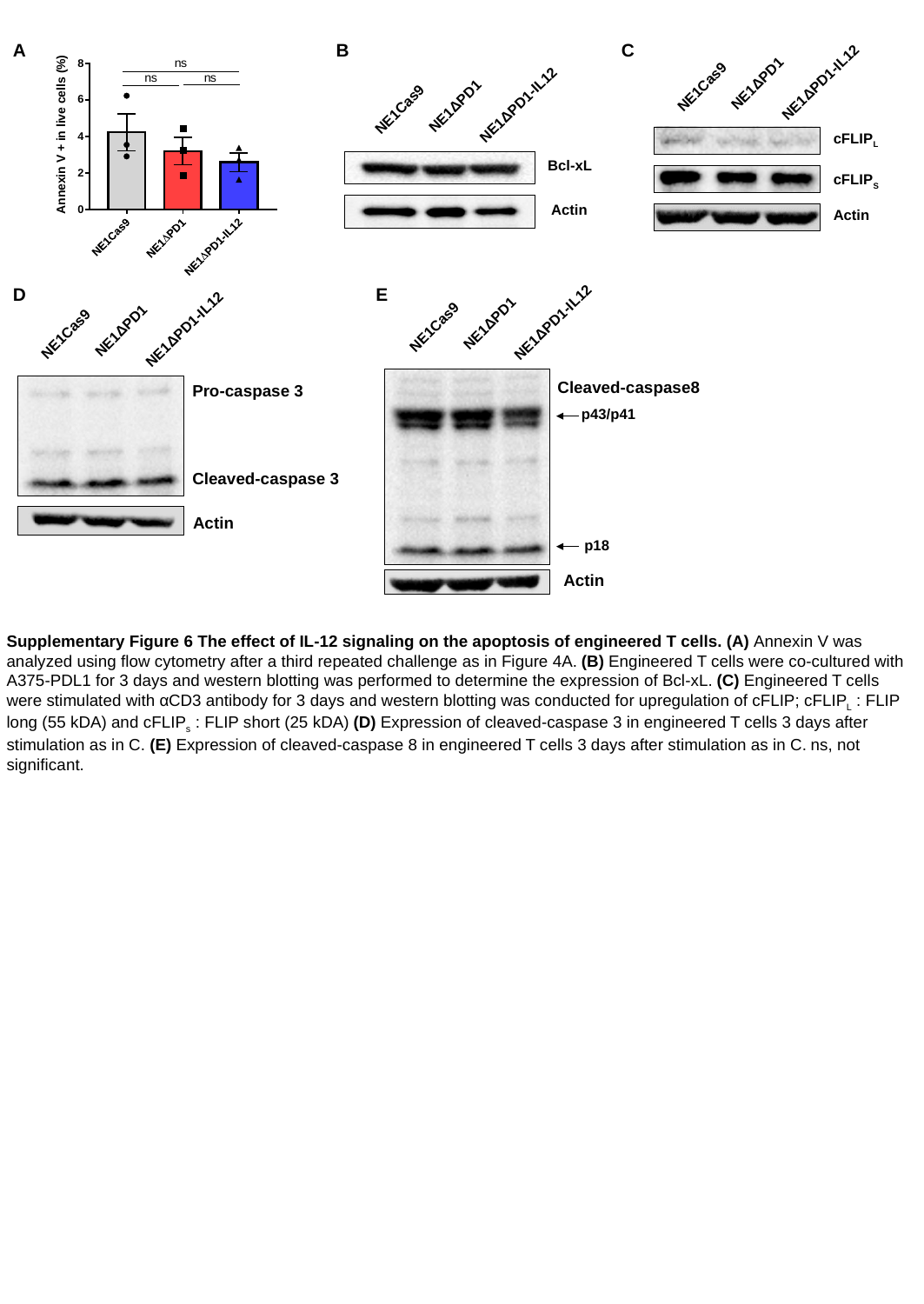

A
B
C
NE1ΔPD1-IL12
NE1Cas9
NE1ΔPD1
cFLIPL
cFLIPS
Actin
NE1ΔPD1-IL12
NE1ΔPD1
NE1Cas9
Bcl-xL
Actin
D
NE1ΔPD1-IL12
NE1ΔPD1
NE1Cas9
Pro-caspase 3
Cleaved-caspase 3
Actin
E
NE1ΔPD1-IL12
NE1ΔPD1
NE1Cas9
Cleaved-caspase8
p43/p41
p18
Actin
Supplementary Figure 6 The effect of IL-12 signaling on the apoptosis of engineered T cells. (A) Annexin V was analyzed using flow cytometry after a third repeated challenge as in Figure 4A. (B) Engineered T cells were co-cultured with A375-PDL1 for 3 days and western blotting was performed to determine the expression of Bcl-xL. (C) Engineered T cells were stimulated with αCD3 antibody for 3 days and western blotting was conducted for upregulation of cFLIP; cFLIPL : FLIP long (55 kDA) and cFLIPs : FLIP short (25 kDA) (D) Expression of cleaved-caspase 3 in engineered T cells 3 days after stimulation as in C. (E) Expression of cleaved-caspase 8 in engineered T cells 3 days after stimulation as in C. ns, not significant.

## Slide 5
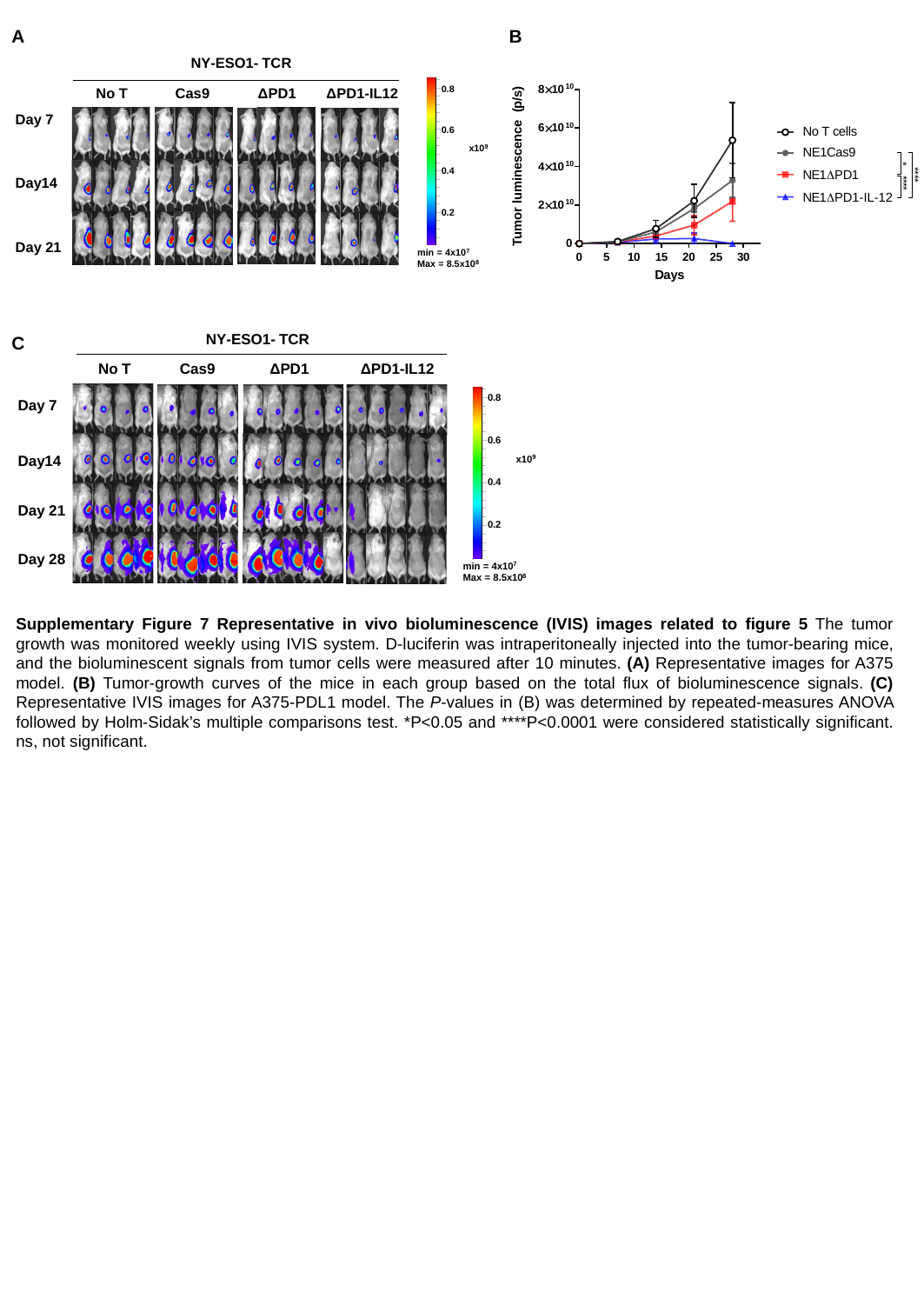

A
B
C
Supplementary Figure 7 Representative in vivo bioluminescence (IVIS) images related to figure 5 The tumor growth was monitored weekly using IVIS system. D-luciferin was intraperitoneally injected into the tumor-bearing mice, and the bioluminescent signals from tumor cells were measured after 10 minutes. (A) Representative images for A375 model. (B) Tumor-growth curves of the mice in each group based on the total flux of bioluminescence signals. (C) Representative IVIS images for A375-PDL1 model. The P-values in (B) was determined by repeated-measures ANOVA followed by Holm-Sidak’s multiple comparisons test. *P<0.05 and ****P<0.0001 were considered statistically significant. ns, not significant.

## Slide 6
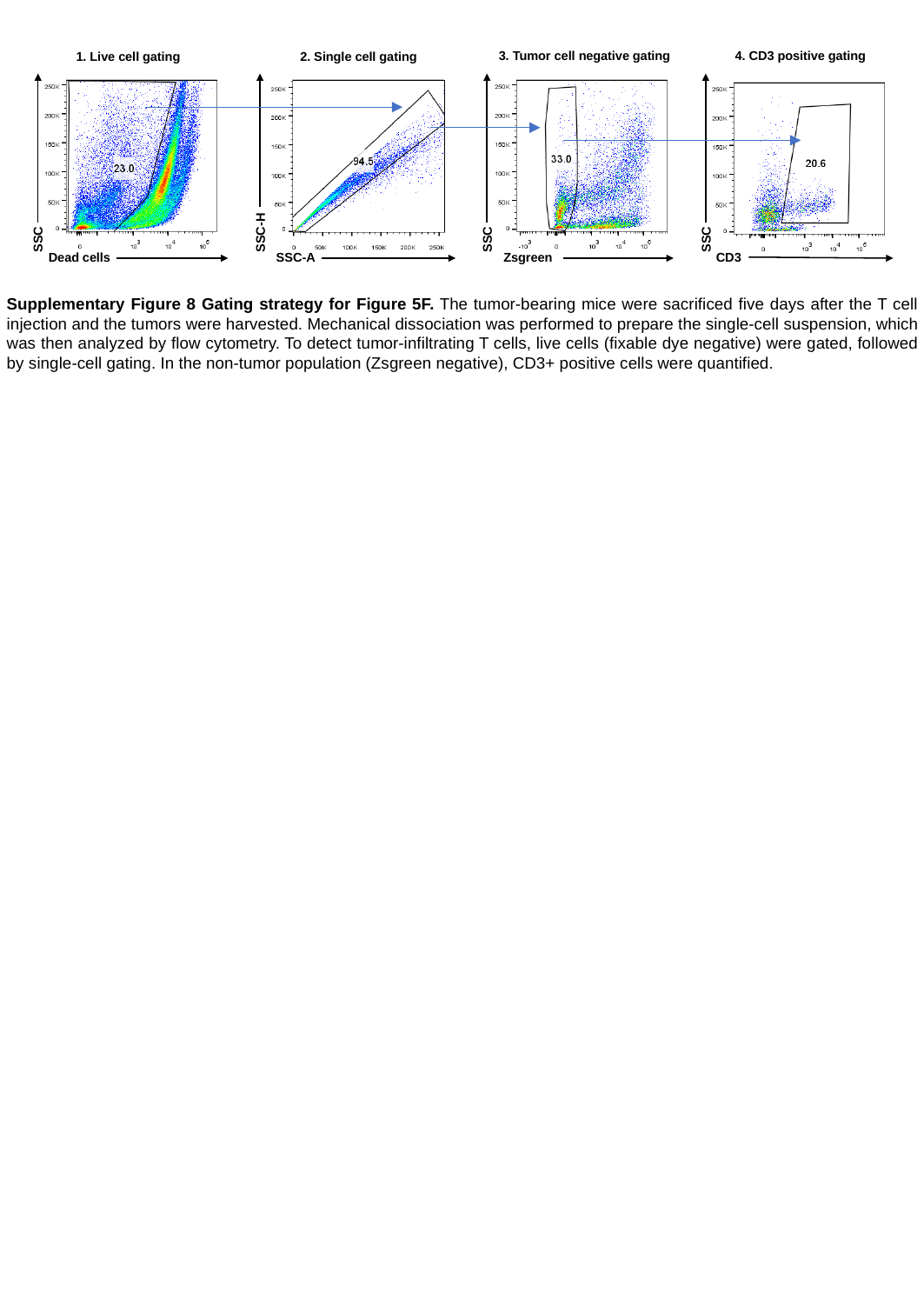

4. CD3 positive gating
3. Tumor cell negative gating
2. Single cell gating
1. Live cell gating
SSC-H
SSC
SSC
SSC
Dead cells
SSC-A
Zsgreen
CD3
Supplementary Figure 8 Gating strategy for Figure 5F. The tumor-bearing mice were sacrificed five days after the T cell injection and the tumors were harvested. Mechanical dissociation was performed to prepare the single-cell suspension, which was then analyzed by flow cytometry. To detect tumor-infiltrating T cells, live cells (fixable dye negative) were gated, followed by single-cell gating. In the non-tumor population (Zsgreen negative), CD3+ positive cells were quantified.
